# Transcriptome-wide analysis of filarial extract-primed human monocytes reveal changes in LPS-induced PTX3 expression levels

**DOI:** 10.1038/s41598-019-38985-x

**Published:** 2019-02-22

**Authors:** B. C. Buerfent, L. Gölz, A. Hofmann, H. Rühl, W. Stamminger, N. Fricker, T. Hess, J. Oldenburg, M. M. Nöthen, J. Schumacher, M. P. Hübner, A. Hoerauf

**Affiliations:** 1 Institute for Medical Microbiology, Immunology and Parasitology, University Hospital of Bonn, Bonn, Germany; 2 Institute of Human Genetics, University of Bonn, Bonn, Germany; 3 Department of Genomics, Life & Brain Center, University of Bonn, Bonn, Germany; 4 Department of Orthodontics, Center of Dento-Maxillo-Facial Medicine, University of Bonn, Bonn, Germany; 5 Department of Orthodontics and Orofacial Orthopedics, University Hospital of Erlangen, Erlangen, Germany; 6 Institute of Experimental Hematology and Transfusion Medicine, University Hospital of Bonn, Bonn, Germany; 7 German Centre for Infection Research (DZIF), partner site Bonn-Cologne, Bonn, Germany; 8 Center for Human Genetics, University Hospital of Marburg, Marburg, Germany

## Abstract

Filarial nematodes modulate immune responses in their host to enable their survival and mediate protective effects against autoimmunity and allergies. In this study, we examined the immunomodulatory capacity of extracts from the human pathogenic filaria *Brugia malayi* (BmA) on human monocyte responses in a transcriptome-wide manner to identify associated pathways and diseases. As previous transcriptome studies often observed quiescent responses of innate cells to filariae, the potential of BmA to alter LPS driven responses was investigated by analyzing >47.000 transcripts of monocytes from healthy male volunteers stimulated with BmA, *Escherichia coli* LPS or a sequential stimulation of both. In comparison to ~2200 differentially expressed genes in LPS-only stimulated monocytes, only a limited number of differentially expressed genes were identified upon BmA priming before LPS re-stimulation with only PTX3↓ reaching statistical significance after correcting for multiple testing. Nominal significant differences were reached for metallothioneins↑, MMP9↑, CXCL5/ENA-78↑, CXCL6/GCP-2↑, TNFRSF21↓, and CCL20/MIP3α↓ and were confirmed by qPCR or ELISA. Flow cytometric analysis of activation markers revealed a reduced LPS-induced expression of HLA-DR and CD86 on BmA-primed monocytes as well as a reduced apoptosis of BmA-stimulated monocytes. While our experimental design does not allow a stringent extrapolation of our results to the development of filarial pathology, several genes that were identified in BmA-primed monocytes had previously been associated with filarial pathology, supporting the need for further research.

## Introduction

Human filarial nematodes cause chronic infections that persist for several years and lead to debilitating diseases like onchocerciasis and lymphatic filariasis, which belong to the group of neglected tropical diseases^1,2^. Generally, filariae modulate the host’s immune response to enable their long-term survival in their hosts. Accordingly, patients infected with filariae develop type 2 immune responses that are characterized by increased production of type 2 cytokines and immunoglobulins including IL-4, IL-5, IL-13, IgE, IgG4, and an eosinophilia. Antigen presenting cells like macrophages are modulated in this context as well. Monocytes from patients infected with the filarial nematode *Brugia malayi* display impaired toll-like receptor (TLR) responses and a diminished expression of pro-inflammatory chemokines^3,4^. Comparing the immune response of lymphatic filariasis patients that were microfilariae positive and microfilariae negative revealed that the presence of microfilariae dampens all filarial-specific and bystander responses^5^. Accordingly, *in vitro* experiments showed that stimulation with *B. malayi* microfilariae lysate increases the expression of regulatory markers like interleukin (IL)−10 and PD-L1 on monocytes of non-endemic controls revealing a phenotype that resembles *Wuchereria bancrofti* infected patients without pathology^6^. Similarly, *in vitro* exposure of human monocytes to *B. malayi* microfilariae increases the expression of chemokines that are associated with an alternative activation^7^. Both studies showed that *B. malayi* microfilariae stimulation suppresses the phagocytic capacity of monocytes/macrophages.

Filarial immunomodulation does not only allow the long-term survival of the parasite within its host, but may also benefit the host. Several human and experimental animal studies demonstrated that helminths can protect from allergies and autoimmune diseases by dampening inflammatory immune responses. Accordingly, infections with the rodent filarial nematode *Litomosoides sigmodontis* suppressed asthma symptoms in a murine asthma model^8^, protected nonobese diabetic (NOD) mice from the onset of type 1 diabetes in a transforming growth factor (TGF)β dependent manner^9^ and improved glucose tolerance in diet-induced obese mice^10^. Chronic infection with *L. sigmodontis* had also a beneficial effect on *Escherichia coli* (*E. coli)*-induced sepsis in mice by reducing exacerbated inflammation and improving bacterial clearance, therefore increasing sepsis survival^11^. Importantly, subsequent *E. coli*-induced immune paralysis was not worsened by filarial infection^12^. The *L. sigmodontis*-mediated protective effect against an *E. coli*-induced sepsis was due to the functional reprogramming of macrophages, which was recapitulated by priming of murine macrophages with a crude extract of *L. sigmodontis* adult worms (LsAg), reducing macrophage activation upon a subsequent LPS challenge and improving their phagocytic capacity^11^. Such protective immune responses in the absence of infections with living filariae were also induced by the administration of LsAg and delayed the onset of type 1 diabetes in NOD mice and improved glucose tolerance^10,13,14^.

Several filariae-derived antigens and molecules were identified that modulate adaptive and innate immune responses^15,16^. The probably best-described filarial-derived molecule up to date is the excretory-secretory product of *Acanthocheilonema viteae -* ES-62. This molecule suppresses e.g. LPS-induced macrophage responses^17^ and administration of ES-62 ameliorates collagen-induced arthritis, systemic lupus erythematosus and lupus-associated accelerated atherosclerosis^18–20^. In case of collagen-induced arthritis, this was in part accomplished by modulating Th17 responses^21^. Additional helminth-derived products that modulate macrophage responses include chitohexaose, a filarial glycoprotein, which induces arginase 1 and IL-10 production in macrophages and protects mice from endotoxemia^22^. Filariae also produce cystatins, cysteine protease inhibitors, which interfere with antigen presentation^23^, reduce HLA-DR and CD86 expression on human monocytes and induce IL-10 production by macrophages^24,25^. Similar to ES-62, treatment with cystatins protects against a variety of diseases, including allergies^26,27^ and gut inflammation^28–30^. Moreover, exosome-like vesicles secreted by *B. malayi* L3 larvae as well as by rodent filarial nematodes *Heligmosomoides polygyrus* and *L. sigmodontis* contain small RNAs (miRNA and Y RNAs) that reveal immunomodulatory capacity by e.g. suppressing type 2 innate immune responses and by polarizing macrophages^31,32^.

Microarray technology allows the genome-wide unsupervised analysis of gene expression changes upon stimulation in different cell types or organisms. Using such an approach, several studies investigated the gene expression within filariae. Those studies investigated the impact of anti-*Wolbachia* chemotherapy on the gene expression of the rodent filaria *L. sigmodontis*^33^ and *B. malayi* using the *B. malayi* array and Filarial Nematode Oligonucleotide Array-Version 2, respectively^34,35^. Additional analysis compared the gene expression of *B. malayi* L3 larvae during the transmission from the arthropod vector to the mammalian host^36,37^ and microfilariae of *B. malayi* and *B. pahangi*^38^. Such microarrays were further used to investigate stage and gender specific gene expression in *B. malayi*^36,39,40^. In regard to immune responses to filariae, the gene expression of human epithelial explant cells in response to *B. malayi* L3 larvae was investigated by microarrays^41^. Similarly, the impact of *B. malayi* L3 larvae on *in vitro* generated DCs and macrophages was analyzed by microarrays, revealing a minimal change in the gene expression of DCs or Langerhans cells and an inflammatory response of macrophages against the L3 larvae^42,43^. An inhibitory effect was observed in DCs co-cultured with *B. malayi* microfilariae^44^.

As previous studies indicate that filariae and extracts obtained from filariae modulate the host’s immune response, but several microarray approaches often revealed a quiescent response of innate immune cells towards the L3 larvae and microfilariae, we performed a transcriptome-wide expression analysis of the immunomodulation of filarial adult worm extracts and their impact on TLR-induced monocyte responses. Therefore, we performed a transcriptome-wide expression analysis of more than 47.000 transcripts to elucidate the immunomodulatory capacity of *B. malayi* female adult worm extract (BmA) on a subsequent LPS stimulation in monocytes of healthy, non-endemic humans. Understanding the underlying mechanisms that determine immunomodulation by filariae may lead to new strategies to control filarial disease and pathology and may further provide a better understanding of filariae-induced protection against immune diseases that are caused by a dysregulated immune response as in autoimmune and allergic diseases.

## Results

### Transcriptional changes after stimulation with *B. malayi* extract and LPS in monocytes

**Figure 1:**
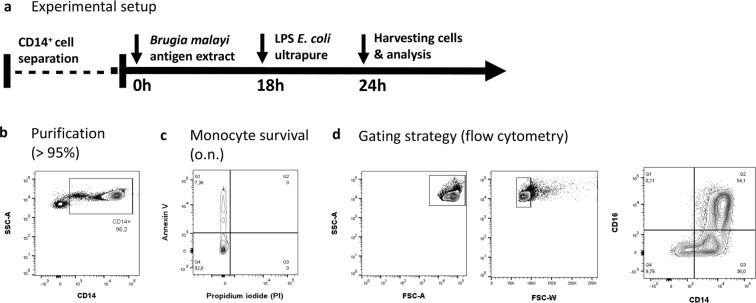
Experimental design and human monocyte characterization. (**a**) Experimental design. Purified CD14^+^ peripheral human blood monocytes were stimulated with *B. malayi* crude antigen extract (BmA) and incubated for 18 h followed by LPS re-stimulation for an additional 6 hours. Afterwards, cells were harvested and stored for RNA preparation. Respective controls received no stimulation or were stimulated with BmA or LPS alone. (**b**) Purity of CD14^+^ monocytes after CD14^+^ bead separation. (**c**) Analysis of monocyte survival after overnight incubation based on Annexin V and Propidium iodide negativity. (**d**) Gating strategy to identify CD14^+^ CD16^+^ and CD14^+^ CD16^-^ subpopulations (SSC: side scatter; FSC: forward scatter).

**Figure 2:**
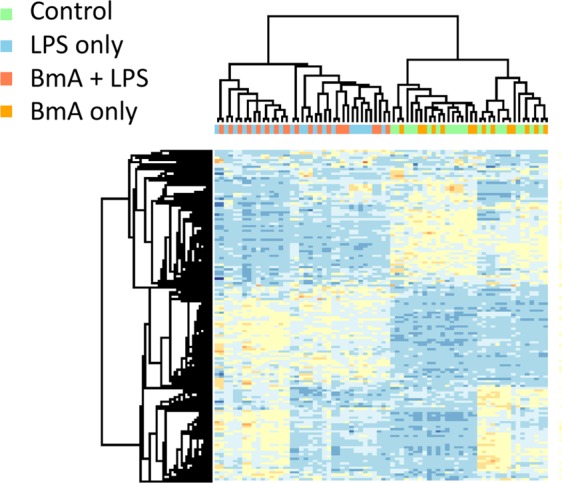
Cluster analysis of most variable genes. Unsupervised hierarchical clustering of the genes with the highest variance (coefficients of variation >5%, n = 1,269) obtained from monocytes stimulated with BmA and/or *E. coli* LPS as well as unstimulated controls. Signal intensities were z-transformed before clustering and clusters for probes (rows) and samples (columns) were calculated using Pearson correlation as distance measurement and average of each cluster for cluster linkage. Color scale represents the mean standard deviant, blue indicates that the signal is below the mean signal intensity of the probe set across the data set, and red indicates that the signal is above the mean signal intensity of the probe set across the data set.

To characterize the modulatory effect of filarial extract in the presence or absence of LPS restimulation on the gene expression profile, monocytes from healthy male subjects were stimulated with (i) *E. coli* LPS, (ii) BmA alone (iii) BmA followed by LPS *E. coli* restimulation (Fig. 1a). Untreated monocytes served as controls. Monocyte purity after separation, overnight survival and expression of CD14 and CD16 after stimulation were analyzed by flow cytometry (Fig. 1b–d). After transcriptome-wide expression profiling using Illumina HT12v4 bead arrays, unsupervised hierarchical cluster analysis of the most variable genes revealed a high correlation between unstimulated controls and BmA-only stimulated as well as between BmA-primed and LPS *E. coli*-restimulated and LPS *E. coli*-only stimulated controls (Fig. 2).

#### Differentially expressed genes after *B. malayi* crude extract stimulation

Differentially expressed genes between unstimulated controls and BmA-only stimulated cells did not withstand correction for multiple testing (BmA vs. control). However, 42 transcripts covering 39 genes were induced by BmA at nominal significance (*P* ≤ 0.05) and a FC ≥1.5 (Supplementary Figure S1, Supplementary Table S1). Genes of the SNOR- and RNU-family were down-regulated by BmA-only stimulation while members of the metallothionein family were up-regulated. In addition, immune-related genes including indoleamine 2,3-dioxygenase 1 (IDO1)↑, TNF alpha induced protein 6 (TNFAIP6)↑, leukocyte immunoglobulin like receptor A3 (LILRA3)↑, C-C motif chemokine ligand 23 (CCL23)↑, and signal transducer and activator of transcription 1 (STAT1)↑ (all up-regulated) as well as CD52↓, albumin (ALB)↓, ribonuclease P RNA component H1 (RPPH1)↓, and Fc fragment of IgE receptor Ia (FCER1A)↓ (all down-regulated) differed significantly upon BmA stimulation.

#### Differentially expressed genes after LPS *E. coli* and BmA + LPS *E. coli* stimulation

**Figure 3:**
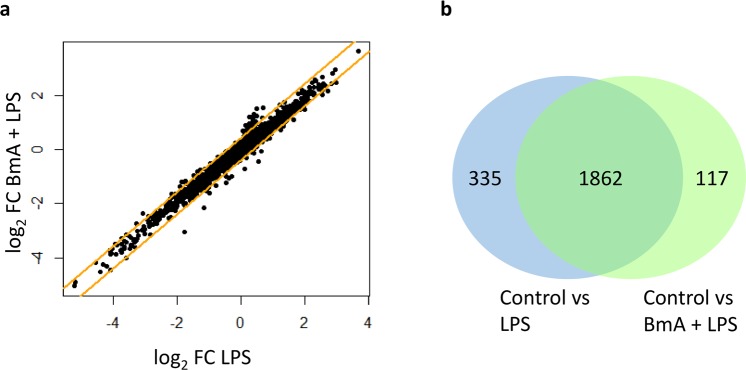
Genome-wide transcriptional changes in human CD14^+^ monocytes after stimulation with *B. malayi* extract plus LPS restimulation and LPS-only stimulation versus unstimulated controls. (**a**) Logarithmic fold-change (FC) for LPS versus unstimulated control against the logarithmic FC for *B. malayi* crude extract (BmA) plus LPS restimulation versus unstimulated control. Orange lines indicate a FC ≥1.5. The correlation of both FCs is 0.98. (**b**) Venn diagram of differentially expressed transcripts (FC ≥1.5 and corrected p-value < 0.05) for the comparison of LPS and BmA + LPS versus control.

When comparing the effect of LPS-only stimulated monocytes and pre-exposure to BmA before LPS restimulation to unstimulated controls (LPS vs. control and BmA + LPS vs. control), a high concordance across fold-changes (r² = 0.98, Fig. 3a) and a high overlap of 1,862 differentially expressed genes of LPS (total of 2,215 differentially expressed genes vs. control) and BmA + LPS (total of 1,979 differentially expressed genes vs. control) stimulated monocytes was observed (Fig. 3b, Supplementary Table S2). Most differentially expressed genes were involved in inflammasome activation and immunity (e.g. interleukin 1 beta (IL1B)↑, tumor necrosis factor superfamily member 12 (TNFSF12)↓, interleukin 6 (IL6)↑, prostaglandin-endoperoxide synthase 2 (PTGS2)↑, C-C motif chemokine ligand 19 (CCL19/CKb11/MIP3B)↑, and C-X-C motif chemokine ligand 1 (CXCL1/GRO1/NAP-3)↑) supporting previous findings in LPS stimulated human monocytes^45,46^.

#### BmA priming alters LPS-induced gene expression

**Figure 4:**
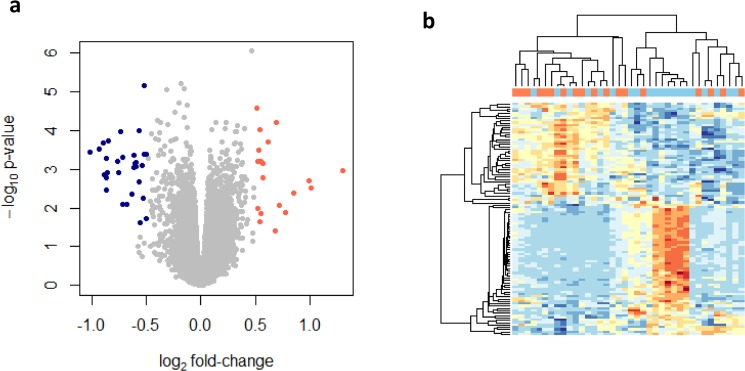
Direct comparison of genome-wide transcriptional changes in human CD14^+^ monocytes after stimulation with *B. malayi* extract plus LPS *E. coli* and LPS alone. (**a**) Volcano plot analysis of *B. malayi* extract (BmA) plus LPS *E. coli* vs. LPS-only induced transcripts. Shown is the logarithmic fold-change (x-axis) against the negative logarithmic p-value (y-axis). Red indicates overexpressed and blue indicates repressed transcripts (p-value < 0.05, FC ≥1.5, n = 49 transcripts). For the complete list of differentially regulated genes and detailed information on the individual probes, see Table 1. (**b**) Hierarchical cluster analysis of differentially expressed genes. Columns represent the individual probes (blue = LPS, orange = BmA + LPS) and rows represent transcripts. In the heatmap, red indicates high relative expression levels and blue indicates low relative expression levels. Cluster analysis was performed using Pearson’s correlation as distance measurement and average linkage.

**Figure 5:**
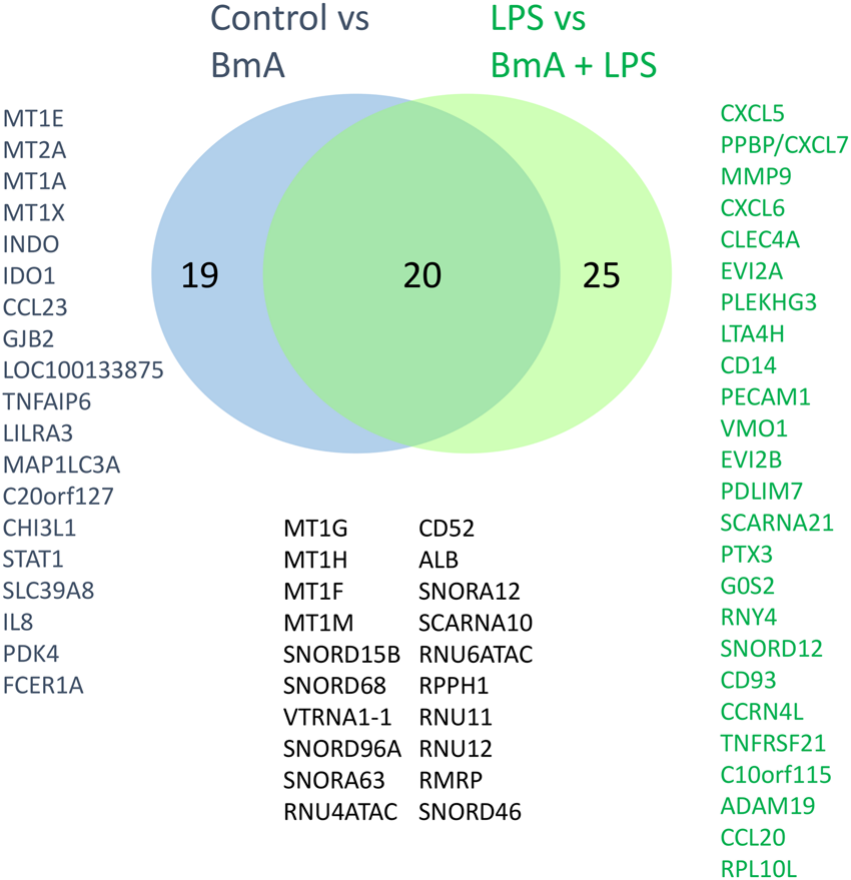
Overlap of genome-wide transcriptional changes in human CD14^+^ monocytes after stimulation with *B. malayi* extract alone and after LPS restimulation in comparison to respective controls. Venn diagram of differentially expressed transcripts (FC ≥1.5 and p-value < 0.05) for the comparison of *B. malayi* extract (BmA) alone versus control and BmA + LPS versus LPS stimulated monocytes.

**Figure 6:**
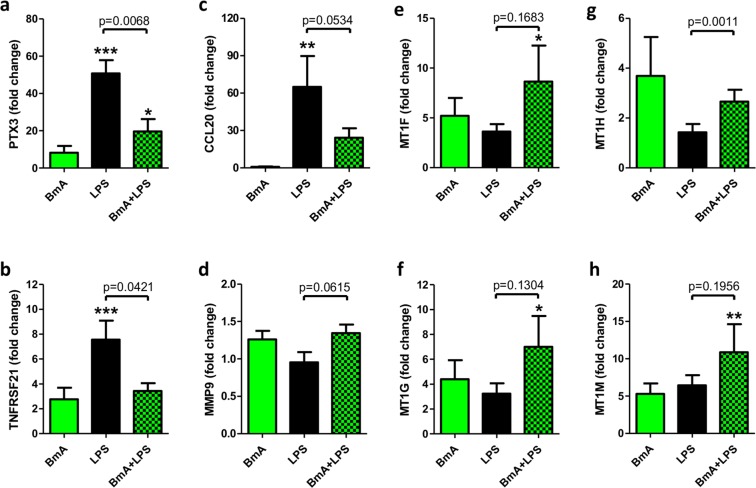
*B. malayi* crude extract priming reduces the LPS-induced gene expression of PTX3, CCL20 and TNFRSF21 and increases the expression of metallothioneins. Fold changes of PTX3 (**a**), TNFRSF21 (**b**), CCL20 (**c**), MMP9 (**d**) as well as of the metallothioneins MT1F (**e**), MT1G (**f**), MT1H (**g**) and MT1M (**h**) of BmA primed and LPS re-stimulated human monocytes and controls (n = 11). The samples were analyzed by quantitative real-time PCR (qPCR) and fold changes in comparison to unstimulated controls are shown (asterisks). The data is presented as mean + SEM. Statistical significance was analyzed by ANOVA followed by Bonferroni Comparison Test (*p < 0,05, **p < 0,01, ***p < 0,01; compared to unstimulated controls) and by paired t-test.

To elucidate transcriptional changes by BmA priming on LPS responses, samples stimulated with BmA followed by LPS *E. coli* restimulation were directly compared to LPS-only stimulated monocytes. Notably, BmA induced 19 up-regulated and 30 down-regulated transcripts mapping to 45 genes with nominal significance and a FC ≥1.5 (Fig. 4, Supplementary Table S3). Only pentraxin 3 (PTX3/TSG-14)↓, a member of the pentraxins superfamily and a multifunctional soluble pattern recognition receptor, remained significantly reduced after multiple test correction. In addition to the increased expression of metallothionein family members and repression of members of the SNOR C/D box, SNOR H/ACA box and RNU-family genes, members of the CXC chemokine family CXCL5/ENA-78↑, CXCL6/GCP-2↑, and CXCL7/NAP-2/PPBP↑, as well as matrix metalloproteinase MMP9↑ were up-regulated. In contrast, ALB↓, TNFRSF21↓, and CCL20/MIP3α↓ were down-regulated by BmA priming before LPS *E. coli* stimulation at a nominal significance. A detailed comparison of genes differentially expressed by BmA alone vs. unstimulated and BmA priming followed by LPS *E. coli* restimulation vs. LPS-only stimulation is displayed in Fig. 5. qPCR analysis confirmed some of these findings and showed a suppression of LPS-induced PTX3 (p < 0.01) and TNFRSF21 (p < 0.05) in BmA primed monocytes compared to LPS-only stimulated monocytes (Fig. 6a,b). CCL20 (Fig. 6c) showed a similar trend towards reduced expression, but this difference did not reach statistical significance (p = 0.053). Additionally, MTH1 (p < 0.01; Fig. 6g) was increasingly expressed in BmA-primed and LPS re-stimulated monocytes compared to LPS-only stimulated monocytes with a similar, non-significant trend observed for MMP9 (p = 0.061; Fig. 6d). Furthermore, BmA-primed and LPS re-stimulated monocytes revealed a significantly upregulated expression of metallothionein family members MT1F, MT1G, MT1M compared to unstimulated controls (Fig. 6e,f,h). This highlights that some of the BmA modulated gene expression is only observed following a pro-inflammatory stimulus like LPS.

### Time-course data

In order to analyze, whether an extended period of BmA stimulation increases the number of differentially expressed genes, stimulations with BmA for 42 h and 48 h were performed. The stimulation with BmA for 42 h and 48 h resulted in 7 and 4 differentially expressed genes, respectively, and was therefore below the number of differentially expressed genes after 24 h of stimulation (see Supplementary Fig. S2). Moreover, genes that were identified after prolonged exposure to BmA were also present at the shorter stimulation time.

This reduced number of differentially expressed genes at longer periods of *in vitro* stimulation were probably due to the short lifespan of classical monocytes in the peripheral blood^47,48^ and the kinetic of transcriptional changes. Accordingly, a previous study on LPS responses in macrophages demonstrated that distinct phases of gene expression exist, with primary response genes been differentially expressed at 0.5–2 h post LPS stimulation (transcription factors that are constitutively expressed, e.g. NFκB, IRFs), secondary response genes at 2–8 h (transcription factors that are de novo synthesized, e.g. C/EBPδ, which could function as master regulators) and chromatin remodeling occurring later on during macrophage differentiation^49^.

### CD86 and HLA-DR expression on human CD14^+^ monocytes and secretion of CXCL5 and CXCL6 after exposure to BmA and LPS

**Figure 7:**
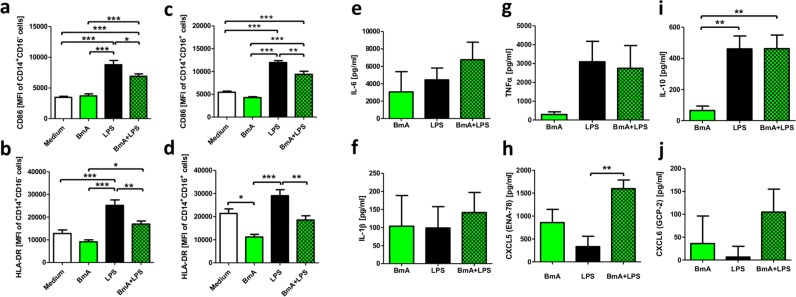
*B. malayi* extract priming reduces CD86 and HLA-DR expression of LPS-stimulated human CD14^+^ monocytes and triggers CXCL5 release. Mean fluorescence intensity (MFI) of CD86 and HLA-DR of human CD14^+^CD16^−^ and CD14^+^CD16^+^ monocytes stimulated with *B. malayi* extract (BmA) and/or *E. coli* LPS as well as unstimulated controls (**a–d**; n = 8 per group). Concentrations of IL-6 (**e**), IL-1β (**f**), TNF (**g**), CXCL5 (**h**), IL-10 (**i**), and CXCL6 (**j**) in the supernatant after stimulation (n ≥ 13 per group). Baseline concentrations of unstimulated controls were subtracted for each donor. Data is presented as mean + SEM and analyzed for statistical significance using ANOVA followed by Bonferroni Comparison Test (*p < 0,05, **p < 0,01, ***p < 0,01).

To confirm the effects obtained from the transcriptome-wide expression profiles (see Supplementary Tables S1,S3) and to further analyze the effect on the protein level, cells were analyzed by flow cytometry for their expression of the extracellular activation markers CD86 and the MHC class II cell surface receptor human leukocyte antigen-DR (HLA-DR) (Fig. 7a–d). CD86 as well as HLA-DR expression on CD16^−^CD14^+^ classical and CD16^+^CD14^+^ non-classical/intermediate monocytes was not induced above baseline after 24 h of BmA stimulation alone. Priming of BmA followed by LPS re-stimulation led to a significantly lower expression of CD86 and HLA-DR on both monocyte subsets compared to LPS-only stimulated controls (Fig. 7a–d). Additionally, the cytokines IL-1β, IL-6, IL-10, and TNF as well as the chemokines CXCL5 and CXCL6 were quantified in the supernatant of the stimulated cells and controls (Fig. 7e–j). According to the transcriptome data, these cytokines and chemokines were secreted after exposure to BmA as well as LPS and no differences in the levels of IL-1β, IL-6, IL-10, and TNF in dependence of BmA priming were observed. CXCL7/PPBP was also measured by qPCR and ELISA, but differences in gene expression observed by the microarray were not confirmed. In contrast, CXCL5 secretion (Fig. 7h) was significantly and CXCL6 secretion (Fig. 7j) by trend increased in cells that were BmA + LPS stimulated compared to LPS only stimulated controls **(**Fig. 7h, Supplementary Table S3).

#### Pathways analysis

To elucidate biological pathways and implicated diseases, explorative enrichment analyses for KEGG, Gene Ontology (GO), Protein-Protein-Interaction networks (PPI) and associated diseases were performed. In addition, Ingenuity Pathway Analysis (IPA) was applied. As only PTX3 reached statistical significance after correcting for multiple testing, we concentrated on differentially expressed genes upon BmA priming before LPS re-stimulation with nominal significance that were confirmed by an additional method, with a minimum of 3 genes (and 2 confirmed genes) per pathway. Differentially expressed genes between LPS-only and BmA followed by LPS *E. coli* restimulation were clustered in KEGGs “chemokine signaling pathway”, KEGGs “cytokine-cytokine receptor interaction”, and KEGGs “inflammation” (Table 1). Diseases that were associated by KEGGs analysis with differentially expressed transcripts by BmA priming before LPS re-stimulation included “bronchial diseases”, “infections of the respiratory tract” and “rheumatoid arthritis” (Table 1). The immune-modulating activity by BmA priming before LPS re-stimulation was supported by an overrepresentation of chemokine and cytokine pathways in the GO analysis (see Supplementary Fig. S3). Importantly, the GO term “cellular response to zinc ion” mapped to four up-regulated metallothionein genes MT1F, MT1G, MT1H, and MT1M demonstrating a general modulation of these pathways, which is supported by the qPCR results showing a trend for increased MT1F, MT1G and MT1M and statistical significant increased expression of MT1H (Fig. 6e–h). In the PPI analysis of the 45 genes that were differentially expressed upon BmA priming before LPS challenge compared to LPS-only stimulated monocytes, three highly connected modules were found demonstrating a high interaction with other immune-relevant genes as more than 100 possible interaction partners for CXCL5, CXCL6 and CCL20 (see Supplementary Fig. S4), which were increasingly produced by BmA-primed and LPS stimulated monocytes by trend (CCL20, Fig. 6c; CXCL6, Fig. 7j) and reaching statistical significance for CXCL5 (Fig. 7h).

**Table 1:** Identified pathways affected by BmA treatment.

Comparison	Category	#Gene	Symbol	Statistics
BmA + LPS vs. LPS	Cytokine-cytokine receptor interaction	5	**CXCL5**, *CCL20*, **TNFRSF21**, PPBP, *CXCL6*	C = 161; O = 5; E = 0.64; R = 7.80;rawP = 0.0004; adj P = 0.0012
	Rheumatoid arthritis	3	**CXCL5**, *CCL20*, *CXCL6*	C = 71; O = 3; E = 0.28; R = 10.61; rawP = 0.0028; adj P = 0.0028
	Chemokine signaling pathway	4	**CXCL5**, *CCL20*, PPBP, *CXCL6*	C = 145; O = 4; E = 0.58; R = 6.92; rawP = 0.0026; adj P = 0.0028
	Inflammation	8	*MMP9*, **CXCL5**, *CCL20*, **PTX3**, *CXCL6*, CD14, PECAM1, PPBP	C = 278; O = 8; E = 1.11; R = 7.22; rawP = 1.19e-05; adj P = 9.06e-05
	Bronchial Diseases	6	*MMP9*, **CXCL5**, *CCL20*, *CXCL6*, CD14, LTA4H	C = 139; O = 6; E = 0.55; R = 10.83; rawP = 1.71e-05; adj P = 9.06e-05
	Respiratory Tract Infections	6	*MMP9*, **CXCL5**, *CXCL6*, CD14, PECAM1, PPBP	C = 144; O = 6; E = 0.57; R = 10.46; rawP = 2.09e-05; adj P = 9.06e-05
	Infarction	5	*MMP9*, **PTX3**, CD14, PECAM1, LTA4H	C = 117; O = 5; E = 0.47; R = 10.73; rawP = 9.61e-05; adj P = 0.0002
	Arteriosclerosis	5	*MMP9*, **PTX3**, CD14, PECAM1, PPBP	C = 121; O = 5; E = 0.48; R = 10.37; rawP = 0.0001; adj P = 0.0002
	Arterial Occlusive Diseases	5	*MMP9*, **PTX3**, CD14, PECAM1, PPBP	C = 123; O = 5; E = 0.49; R = 10.20; rawP = 0.0001; adj P = 0.0002
	Myocardial Infarction	5	*MMP9*, **PTX3**, CD14, PECAM1, LTA4H	C = 124; O = 5; E = 0.49; R = 10.12; rawP = 0.0001; adj P = 0.0002
	Common Cold	5	*MMP9*, **CXCL5**, *CXCL6*, CD14, PPBP	C = 140; O = 5; E = 0.56; R = 8.96; rawP = 0.0002; adj P = 0.0003
	Bronchiolitis	5	**CXCL5**, *CXCL6*, CD14, PECAM1, PPBP	C = 142; O = 5; E = 0.57; R = 8.84; rawP = 0.0002; adj P = 0.0003
	Myocardial Ischemia	5	*MMP9*, ALB, **PTX3**, CD14, PECAM1	C = 142; O = 5; E = 0.57; R = 8.84; rawP = 0.0002; adj P = 0.0003

Overview of pathways affected by BmA treatment followed by LPS stimulation (BmA + LPS vs. LPS) determined by KEGGs chemokine signaling pathway analysis. Differentially expressed genes (P value < 0.05; FC ≥ 1.5) of monocytes of human non-endemic controls (n = 20). Transcripts that were confirmed by qPCR or ELISA with statistical significance are marked as bold and by trend as italics. Abbreviations: C: number of reference genes in the category; O: number of genes in the gene set and in the category; E: the expected number in the category; R: ratio of enrichment; rawP: p value from hypergeometric test; adjP: p value adjusted for multiple testing.

Based on the IPA analysis BmA pre-exposure before LPS *E. coli* re-stimulation affected several pathways like granulocyte and agranulocyte adhesion and diapedesis (PPBP/CXCL7, PECAM1, CCL20, CXCL5, CXCL6, MMP9), IL-17 in psoriasis, arthritis and signaling in airway cells (CCL20, CXCL5, CXCL6). With at least two affected genes that were confirmed by a second method, the pathway of IL-17F in allergic inflammatory airway diseases (CXCL5, CXCL6) was identified (see Supplementary Table S4).

**Figure 8:**
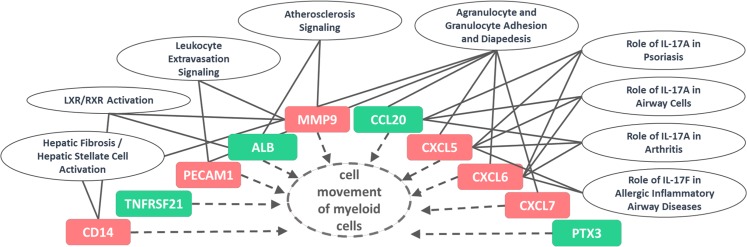
Top 10 canonical pathways affected by *B. malayi* extract priming before LPS stimulation are associated with cell movement of myeloid cells as well as autoimmune and airway diseases. Overview of identified genes effecting the cell movement of myeloid cells and associated pathways. Up-regulated differentially expressed genes (red) and down-regulated genes of BmA primed LPS re-stimulated compared to LPS-only stimulated monocytes are shown (FC ≥1.5 and p-value < 0.05).

In summary, associated diseases enriched for genes that differed upon BmA pre-stimulation included inflammatory diseases, bronchial diseases and respiratory tract infections (Table 1, Supplementary Table S4). Moreover, the IPA analysis indicated that the top 10 differentially expressed transcripts CD14, TNFRSF21, PECAM-1, ALB, MMP9, CCL20, CXCL5–7, and PTX3 are all affecting the movement of myeloid cells and have an impact on several pathways that are related to granulocytes, and inflammatory conditions and diseases (see Fig. 8). Differential expression of those genes were confirmed with statistical significance for TNFRSF21 (Fig. 6b), CXCL5 (Fig. 7h) and PTX3 (Fig. 6a) and by trend for MMP9 (Fig. 6d), CCL20 (Fig. 6c) and CXCL6 (Fig. 7j).

### BmA stimulation reduces apoptosis of monocytes

**Figure 9:**
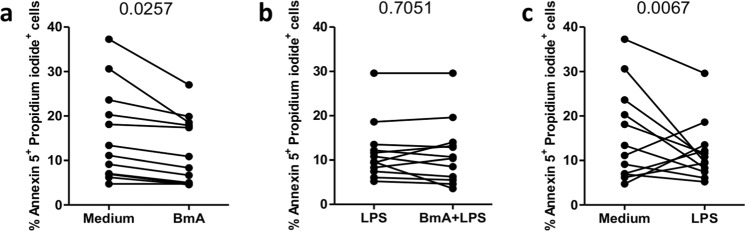
BmA treatment reduces apoptosis of human monocytes. The frequency of Annexin V^+^ Propidium iodide^+^ cells after BmA-only stimulation (**a**), LPS-only stimulation (**b**) and in combination of BmA-priming and LPS stimulation of twelve donors is shown (**c**). The data were analyzed by paired t test for significance.

As metallothioneins were induced upon BmA stimulation and were previously described to prevent apoptosis^50^, we investigated the impact of BmA stimulation on apoptosis. The frequency of Annexin V^+^ Propidiumiodide^+^ apoptotic cells was reduced by BmA treatment compared to unstimulated controls (Fig. 9a). Further explorative IPA® analysis revealed ten apoptosis pathway-related transcripts (ALB↓, CHI3L1↑, CXCL8↑, FCεR1A↓, IDO1↑, MAP1LC3A↑, MT1A↑, MT1F↑, MT2A↑, and STAT1↑) upon BmA-only stimulation compared to controls. However, apoptosis of monocytes following LPS stimulation was not altered by pre-stimulation with BmA **(**Fig. 9b**)**, as LPS-only stimulation significantly reduced apoptosis (Fig. 9c).

## Discussion

Parasitic helminths modulate inflammatory immune responses in their host^51^. The beneficial impact of filarial nematodes and their products on inflammatory diseases was previously shown in the murine model of *L. sigmodontis*, which mediated protection against *E. coli*-induced sepsis^11^ and metabolic disorders like diabetes^13,52^. Similarly, several filarial nematode derived proteins like the recombinant *B. malayi* cystatin (rBmCys), the excretory/secretory protein ES-62 from *Acanthocheilonema viteae* but also glycans from *Fasciola hepatica* were described to modulate immune responses and to possess therapeutic potentials against inflammatory diseases^29,53–57^. Previous microarray analysis were focused on the changes in the gene expression within different filarial stages and genders as well as the impact of anti-*Wolbachia* therapy^33–35^. Concerning the impact on cellular immune responses, such microarray analysis were used for the interaction of DCs and macrophages with microfilariae and L3 larvae revealing only minor changes in the gene expression^42,43,58^. In order to investigate whether gene expression changes are more prominent in response to adult filarial worms and to characterize the modulatory potential of filariae and their products on TLR responses, we used a transcriptome-wide expression profiling of 47.000 transcripts comparing the gene expression of monocytes stimulated with BmA, LPS, and BmA before LPS re-stimulation. Whereas LPS-only stimulation induced ~2200 differentially expressed genes, confirming previous results^45^, BmA stimulation had only a minor impact on the gene expression and was therefore in line with previous microarray analysis investigating the impact of microfilariae and L3 larvae on the gene expression of monocytes and DCs^42,43,58^. In particular, only changes in the soluble pattern recognition receptor PTX3 (pentraxin-3) expression withstood correction for multiple testing, whereas 44 additional genes reached nominal significance in BmA treated and LPS restimulated human monocytes compared to LPS-only stimulation (FC ≥1.5; p ≤ 0.05).

Previous findings showed that PTX3 is altered by mediators like LPS or IL-1β strongly affecting innate immunity and inflammation^59^. PTX3 plays a crucial role in host defense and is increasingly expressed during septic shock, cardiovascular diseases, obstructive pulmonary disease and atherosclerosis^60–64^. PTX3 is an extrinsic oncosuppressor and epigenetically repressed in different human tumors^65^. In the human THP-1 macrophage cell line recombinant PTX3 was shown to reduce TNF-induced NFκB expression but also HLA-DR and CD86 expression on human monocyte-derived macrophages and to play an essential role in tissue remodeling in mice^66–68^. However, BmA-priming before LPS restimulation resulted in our study in a reduced expression of HLA-DR and CD86 on both CD16^+^ and CD16^-^ monocyte subsets, whereas the expression was induced by LPS stimulation alone, as was previously described^69^. Interestingly, BmA alone did not induce the expression of HLA-DR and CD86, suggesting a subordinated mechanism. This indicates that some of the BmA-induced changes are only observed in the presence of a secondary pro-inflammatory stimulus like LPS. Interestingly, Yadav *et al*. described that the *B. malayi* derived protein Calreticulin (BmCRT) competes with PTX3 among others to bind the first complement component C1q of the human host that can prevent the activation of the classical complement pathway^70^. Accordingly, further studies should investigate the modulation of PTX3 expression and decipher its impact on inflammatory immune responses and pathology development during lymphatic filariasis.

The BmA pre-stimulation further revealed nominal significant transcripts that were in part confirmed by qPCR or ELISA. These transcript included interesting gene families that may affect apoptosis pathways, chemoattraction and thus the development of lymphedema. The members of the metallothionein family were increasingly expressed in BmA-primed and LPS re-stimulated monocytes and have the potential to protect cells and tissues against heavy metal toxicity^71^. Interestingly, metallothioneins are increased in different tumor cell lines and described to prevent chemotherapy-induced apoptosis as a cellular protective mechanism during the treatment of acute leukemia leading to drug resistance^50^. According to the potential anti-apoptotic effect of metallothioneins, we observed a reduced apoptosis after BmA treatment, a phenomenon that was previously described in macrophages of *L. sigmodontis*-infected animals upon *E. coli*-challenge^11^. Explorative IPA analysis further supported such an anti-apoptotic effect of BmA, as increased CHI3L1 and CXCL8 expression were previously shown to protect against apoptosis^72,73^.

Furthermore, matrix metalloproteinase MMP9 was increased due to BmA stimulation. MMP9 is involved in the breakdown of the extracellular matrix, e.g. during reproduction^74^. Moreover, MMP9 is also associated with cancer and metastasis by regulating apoptosis and the allocation of VEGF^75^. Importantly, Anuradha *et al*. demonstrated that in *W. bancrofti* infected individuals with filaria-induced pathology, MMPs and their counterparts, the tissue inhibitor of metalloproteinases (TIMPs), are elevated^76^. Previous studies from our institute demonstrated that single nucleotide polymorphisms (SNPs) in MMP2 are a risk factor to develop lymph edema^77^, suggesting that MMP9 could also be involved in the development of filarial pathology. Similarly, SNPs of the pattern recognition receptors TLR4, NOD1 and NOD2 were shown to be associated with patent lymphatic filariasis^78^. As those receptors are expressed by innate cells including monocytes and trigger inflammatory responses, e.g. NOD1 and NOD2 in response to the bacterial *Wolbachia* endosymbionts of filariae^79^, it can be speculated that monocytes are involved in this process.

In contrast, the expression of noncoding RNAs like the small nucleolar RNA families SNOR C/D box, SNOR H/ACA box as well as RNUs were reduced by BmA priming before LPS re-stimulation. Small nucleolar RNAs (snoRNAs) are involved in ribosomal RNA maturation and can function as microRNA^80,81^. Interestingly, to our knowledge associations to inflammatory or filarial diseases are not known so far, except for SNORD12, which is located in the ZFAS1 gene and a potential marker for breast cancer^82^.

Chemotactic molecules induced by BmA priming before LPS re-stimulation included CXCL5 and CXCL6 which are known to attract neutrophils and are linked to systemic diseases such as cancer and atherosclerosis^83^, suggesting an essential role for modulating inflammation and increasing neutrophil activity^84,85^. In contrast, CCL20 was reduced in BmA treated and LPS stimulated monocytes and possesses chemotactic properties for lymphocytes^86^. Thus, filarial extract exposure may have an impact on the capacity of monocytes to modulate cellular recruitment, which may impact the development of filarial pathology as well as inflammatory disorders.

In summary, our transcriptome-wide profiling of BmA pre-stimulated human monocytes before LPS re-stimulation revealed a reduced PTX3 gene expression. While the results of our experiments do not allow stringent conclusions on the chronic exposure to filarial antigens during filariasis and the development of filarial pathology, we identified several additional genes that have been previously linked with the development of lymphedema, indicating that monocyte exposure to filarial extracts may be involved in the pathogenesis of filarial disease.

## Methods

### Ethics approval and consent to participate

The study was approved by the ethics committee of the University of Bonn (No. 352/13) and all methods were performed in accordance with the relevant guidelines and regulations. Study participants were recruited, informed about the present study and informed consent was obtained from all study participants. Peripheral blood samples were taken after consent to participate were provided and all donors were monitored by the blood transfusion service during and after the blood draw. The patients gave their consent to publish the results anonymously.

### Human study population

20 healthy male volunteers of European origin (Caucasian; 18–35 years) were recruited and peripheral blood samples were taken. Inclusion criteria were at least three generations of grandparents in Central Europe, non-smoker, no acute or chronic infection, no vaccination 4 weeks prior to blood withdrawal and C-reactive protein levels <2.5 mg/dl. Female donors were excluded due to hormonal factors possibly influencing transcriptional expression patterns.

### Monocyte isolation

As previously reported by Gölz *et al*.^87,88^, peripheral blood mononuclear cells were purified using a Ficoll-Plaque density gradient from heparinized whole blood. Afterwards, CD14^+^ monocytes were isolated by magnetic-activated cell sorting using CD14-microbeads (Miltenyi Biotec GmbH, Bergisch Gladbach, Germany) and the AutoMACS Pro Separator system according to the manufacturer’s instruction. Cell purity was determined by flow cytometry. Cells were resuspended in RPMI 1640 media supplemented with GlutaMAX^TM^, 10% heat-inactivated FCS (Gibco® Life Technologies, Waltham, USA), 100 U/ml penicillin, and 100 µg/ml streptomycin (both PAA Laboratories/GE Healthcare, Pasching, Austria).

### Monocyte stimulation

Monocytes were cultured in 96-well round bottom wells at a density of 500,000 cells/well in 100 µl for 2 hours after separation. Afterwards, cells were stimulated with 10 µg/ml BmA for 18 h and re-stimulated for an additional six hours with 200 ng/ml LPS *E. coli* ultrapure (InvivoGen, San Diego, USA). Non-stimulated monocytes and single BmA or LPS stimulated samples served as control (Fig. 1a).

### BmA preparation

Adult *B. malayi* female worms (obtained from NIAID Filariasis Research Reagent Resource Center (FR3)) were washed with sterile PBS and then placed inside a glass mortar. Sterile RPMI 1640 medium was added and the worms were homogenized. Afterwards the extract was centrifuged for 10 min at 300 x g at 4 °C. The supernatant was collected and the protein concentration was determined by Bradford assay, aliquoted and stored at −80 °C until usage. One batch of BmA was used for this study and was confirmed to lack detectable endotoxin levels by QCL-1000™ Endpoint Chromogenic LAL assay (Lonza, Basel, Switzerland). BmA concentrations used in this study did not affect cell viability as was tested by the Colorimetric Cell Viability Kit III (XTT; PromoKine, Heidelberg, Germany).

As previously reported, overnight monocyte survival was checked microscopically by trypan blue staining and confirmed by Annexin V (Apoptosis Detection Kit) and propidium iodide analysis (eBioscience, San Diego, USA) via flow cytometry^87^. After stimulation supernatants were collected, cells were lysed in RLT Plus buffer (QIAGEN, Hilden, Germany) and stored at −80 °C. After applying stringent inclusion criteria (C-reactive protein levels <2.5 mg/dl, monocyte purity >95%), monocytes of 20 individuals were further processed. In additional experiments, monocytes were stimulated after a resting period of 2 h for 42 h and 48 h. Therefore, monocytes were primed for 18 h or 24 h with BmA followed by 24 h or 18 h of LPS stimulation (total 42 h), respectively. Additionally, a long-term treatment of 48 h including a 24 and 42 h priming with BmA followed by 24 or 6 h of LPS re-stimulation was performed (see Supplementary Fig. S2).

### RNA extraction

RNA from lysed monocytes was extracted using the AllPrep DNA/RNA Mini Kit from QIAGEN following manufacturer’s instruction. For quality control RNA concentrations were measured using NanoDrop (PeqLab, Erlangen, Germany) and degradation via Bioanalyzer (RIN 7; Agilent Technologies, Santa Clara, USA).

### Transcriptome analysis

The Illumina TotalPrep RNA Amplification Kit (Life Technologies) was used to amplify and biotinylate the RNA. Whole transcriptome profiling was performed on Illumina’s HT-12v4 bead arrays (Illumina, San Diego, USA) comprising 47.231 transcripts.

Statistical analysis was performed using functions implemented in the statistical software R (version 3.1.0) and Bioconductor packages. Expression data were quantile normalized using the limma package^89^ and subsequently log-transformed. The selected expression probes were then filtered for a perfect or good probe quality, as reported in the Bioconductor package illuminaHumanv4.db^90^. After quality control and filtering, data for 18.452 expression probes from up to 20 individuals were included in the analysis. Differentially expressed probes were calculated using limma^89^ applying a paired design in the ANOVA analysis to identify the contrast between controls and stimulated samples. All nominal *p*-values *P* were calculated using a Student’s t-test and adjusted for multiple testing using Benjamini-Hochberg correction in order to control for false discovery if not reported otherwise. Only probes with a fold-change (FC) ≥1.5 were selected in line with the microarray sensitivity of 1.35. The fold-change was calculated by dividing the mean intensity of the expression of a target gene of one group by the expression intensity of the same gene from the group it was compared with. If this number was < 1, the negative reciprocal was used. The datasets generated during the current study are available in the Annotare, ArrayExpress by EMBL-BI repository (https://www.ebi.ac.uk/fg/annotare/login/).

Hierarchical cluster analysis was performed using the cluster method in R on z-transformed probes. Distances of the samples were calculated using Pearson correlation and clusters were formed by taking the average of each cluster.

### Pathway analysis

Data evaluations were performed using Ingenuity Pathway Analysis (IPA^®^ Systems Inc., Redwood City, USA). Thereby, each gene is represented in a global molecular network designed by information provided by the Ingenuity Pathways Knowledge Base. “Networks” were generated algorithmically based on their connectivity concerning activation, expression and transcription. Molecular relationships between genes are visible as connecting lines between nodes supported by published data stored in Ingenuity Pathways Knowledge Base and/or PubMed. In addition, enriched GO and KEGG pathways, Protein-Protein-Interaction networks and associated diseases were calculated using the WEB-based GEne SeT AnaLysis Toolkit (WebGestalt^91^) using all analyzed probes (n = 18,452) as the background reference, a minimum of 3 genes per category and a FDR of 1% as the threshold for significance.

### Flow cytometry, immunoassays and quantitative real-time PCR assays (qPCR)

For analysis of monocyte activation, the cell suspension was centrifuged at 300 g for 10 min. The cells were incubated in PBS/1% BSA including 0.1% IgG from rat serum for 30 min (Sigma-Aldrich, St. Louis, USA). Afterwards, the cells were stained with CD14 FITC (Clone: 62D3), CD16 APC (Clone: eBioCD16), HLA-DR PerCP-Cy5.5 (Clone: LN3, eBioscience), and CD86 PE (Clone: 2331 (FUN-1), BD Bioscience, San Diego, USA). Flow cytometry was performed using BD FACS Canto followed by analysis with FACS Diva software (BD Biosciences) and FlowJo V10 software (FLOWJO LLC, Ashland, USA). The cytokine and chemokine concentrations in the cell culture supernatant were measured by ELISA (CXCL5/ENA-78 and CXCL6/GCP-2) using SpectraMAX 190 and SoftMAX Pro 6.5 software (Molecular Devices, Sunnyvale, USA) or by Multiplex Immunoassays (tumor necrosis factor (TNF)α, IL-1β, IL-6, IL-10, all R&D Systems, Minneapolis, USA) using a xMAP MAGPIX system according to the manufacturer’s instruction (Luminex Corporation, Austin, USA). For quantitative real-time PCR (qPCR) cDNA synthesis was performed using the iScript® Advanced cDNA Kit according to the manufacturer instructions (BioRad, Hercules, USA). Gene expression of PTX3 (qHsaCED0021520), TNFRSF21 (qHsaCID0015418), CCL20 (qHsaCID0011773), MMP9 (qHsaCID0011597), MT1F (qHsaCED0057490), MT1G (qHsaCED0020789), MT1H (qHsaCED0048507), MT1M (qHsaCED0048508) and GAPDH (qHsaCED0038674) were measured using BioRad PrimePCR^TM^ SYBR® Green Assay with SsoAdvanced^TM^ Universal SYBR® Green Supermix according to the manufacturer instructions. The fold changes (2^−∆∆C^_T_) were calculated after Schmittgen and Livak^92^.

The secreted proteins, surface marker expression profiles and qPCR results were analyzed by ANOVA followed by Bonferroni Comparison Test or by Paired t test using GraphPad Prism Version 5.0 (GraphPad Software, San Diego, USA). Data are shown as mean with SEM and *p*-value of 5% was considered as statistical significant.

Parts of the methological descriptions are published within the doctoral thesis from BCB^93^.

## Supplementary information

Supplementary Information

Supplementary Table S2

## Data Availability

All datasets generated during the current study are online accessible in the ArrayExpress database at EMBL-EBI (www.ebi.ac.uk/arrayexpress) under accession number E-MTAB-7608.
