# Supplementary material for: Transcriptome-wide analysis of filarial extract-primed human monocytes reveal changes in LPS-induced PTX3 expression levels
Source: Sci Rep. 2019 Feb 22;9:2562. doi: 10.1038/s41598-019-38985-x (PMC6385373; doi:10.1038/s41598-019-38985-x)
Supplement: Supplementary file 1 — Supplementary Information [file 41598_2019_38985_MOESM1_ESM.docx]

**Transcriptome-wide analysis of filarial extract-primed human monocytes reveal changes in LPS-induced PTX3 expression levels**

**Supplementary Information**

Buerfent BC^1,2,3,8^, Gölz L^4,5^, Hofmann A^1,2,3^, Rühl H^6^, Stamminger W^1^, Fricker N^2,3^, Hess T^2,3,8^, Oldenburg J^6^, Nöthen MM^2,3^, Schumacher J^2,3,8^, Hübner MP^1*+^, Hoerauf A^1,7+^

^1^Institute for Medical Microbiology, Immunology and Parasitology, University Hospital of Bonn, Bonn, Germany

^2^Institute of Human Genetics, University of Bonn, Bonn, Germany

^3^Department of Genomics, Life & Brain Center, University of Bonn, Bonn, Germany

^4^Department of Orthodontics, Center of Dento-Maxillo-Facial Medicine, University of Bonn, Bonn, Germany

^5^Department of Orthodontics and Orofacial Orthopedics, University Hospital of Erlangen, Erlangen, Germany

^6^Institute of Experimental Hematology and Transfusion Medicine, University Hospital of Bonn, Bonn, Germany

^7^German Centre for Infection Research (DZIF), partner site Bonn-Cologne, Bonn, Germany

^8^Center for Human Genetics, University Hospital of Marburg, Marburg, **
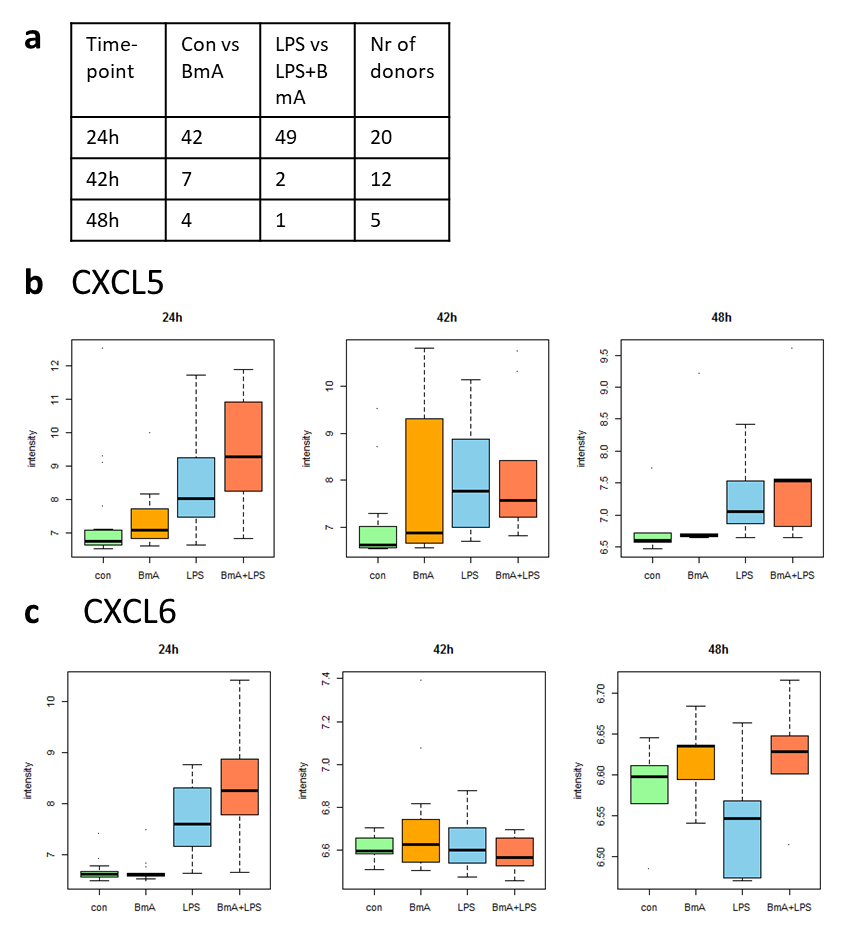
**

**Figure S4: Time-course analysis**

Comparison of the time-points 24 h, 42 h and 48 h. For the 42 h time-point, the monocytes received BmA-stimulation for 18 h and re-stimulation with LPS *E. coli* for additional 24 h. The 48 h time-point tested stimulation with BmA for 24 h and subsequent re-stimulation with LPS for 24 h. BmA-only controls were stimulated for 42 h or 48 h total. Shown is in (a) the number of differentially expressed genes at each time-point for the comparison of controls vs BmA and the comparison of LPS vs LPS+BmA and the expression profile for the candidate genes CXCL5 (b) and CXCL6 (c) at time-points 24 h, 42 h and 48 h.

Germany

^+^authors contributed equally

**
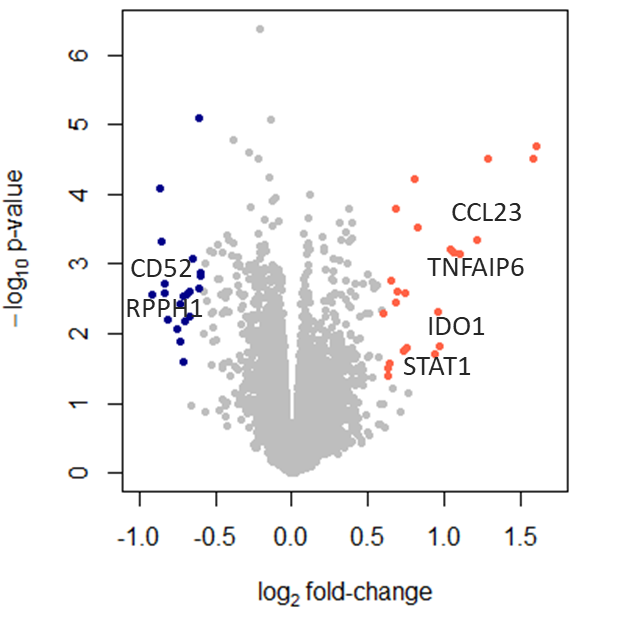
**

**Figure S1: Genome-wide transcriptional changes induced by *B. malayi* extract stimulation compared to unstimulated human CD14^+^ monocytes.** Volcano plot analysis of *B. malayi* antigen induced transcripts. Shown is the logarithmic fold-change (x-axis) against the negative logarithmic p-value (y-axis). Red indicates overexpressed and blue indicates repressed transcripts (p-value < 0.05, FC ≥ 1.5, n = 42 transcripts). Immune-related genes are highlighted. For the complete list of differentially regulated genes and detailed information on the individual probes, see Table S1 in the supplemental material.

**
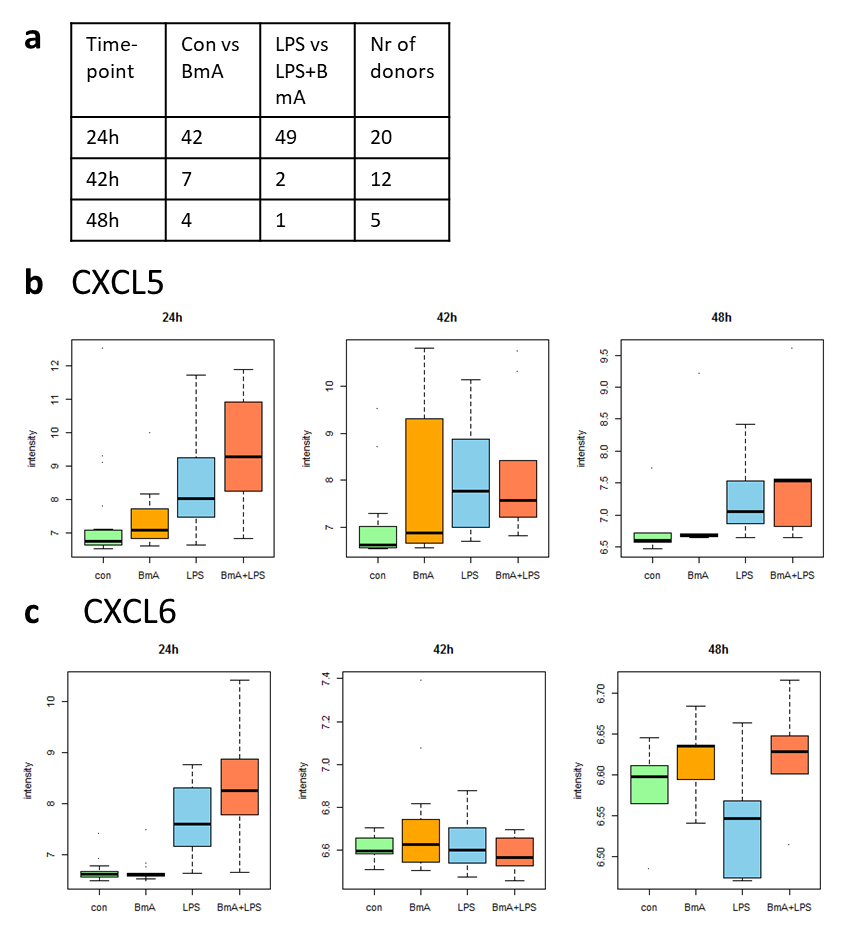
**

**Figure S2: Time-course analysis**

Comparison of the time-points 24 h, 42 h and 48 h. For the 42 h time-point, the monocytes received BmA-stimulation for 18 h and re-stimulation with LPS *E. coli* for additional 24 h. The 48 h time-point tested stimulation with BmA for 24 h and subsequent re-stimulation with LPS for 24 h. BmA-only controls were stimulated for 42 h or 48 h total. Shown is in (a) the number of differentially expressed genes at each time-point for the comparison of controls vs BmA and the comparison of LPS vs LPS+BmA and the expression profile for the candidate genes CXCL5 (b) and CXCL6 (c) at time-points 24 h, 42 h and 48 h.

**
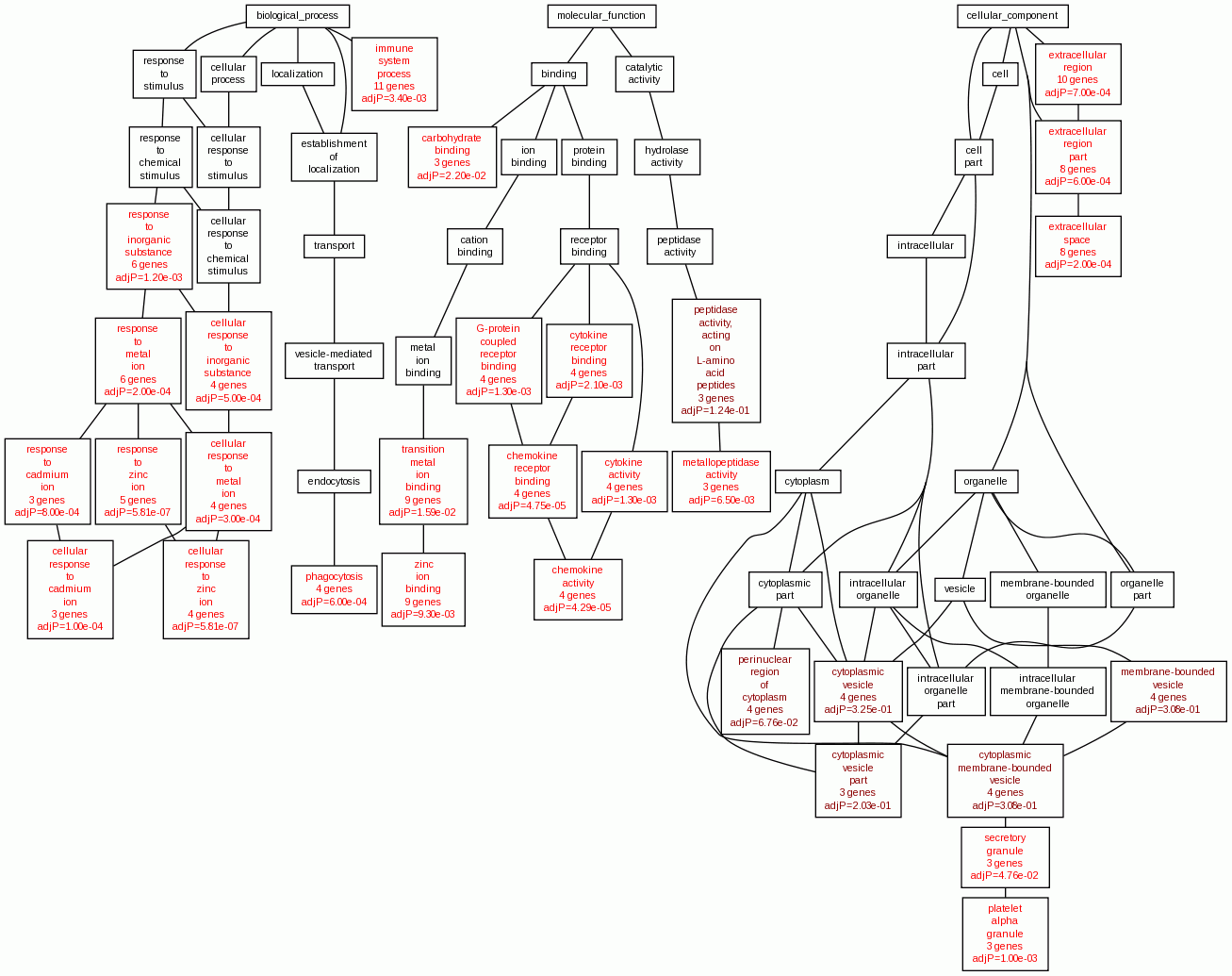
**

**Figure S3: Gene Ontology enrichment analysis.** Gene Ontology (GO) enrichment analysis for differentially expressed genes between *E. coli* LPS versus *B. malayi* extract plus LPS *E. coli* stimulated monocytes (n=45 genes). Analysis was performed using WebGestalt with a minimum of 3 genes per GO term.

**
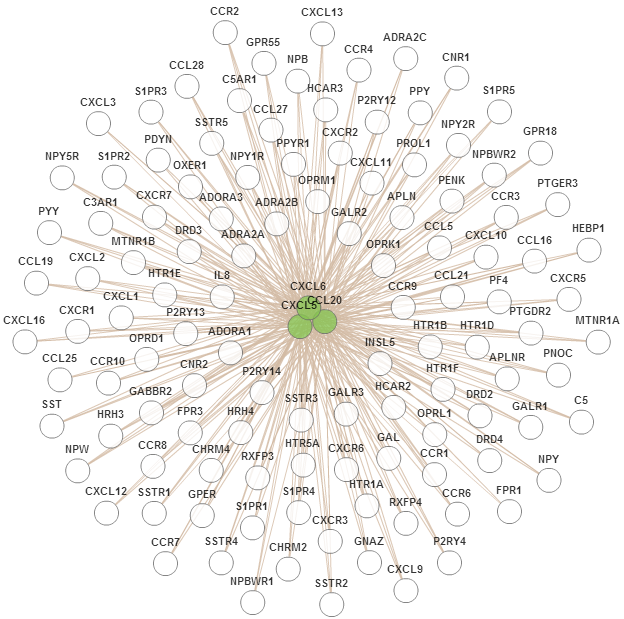
**

**Figure S4: Protein-Protein Interaction analysis.** The Protein-Protein Interaction analysis of differentially expressed genes of *B. malayi* extract + LPS and LPS-only stimulated monocytes identified three highly connected modules. Module 62 including the differentially expressed genes CXCL5, CXCL6 and CCL20 and their interacting genes is shown.

**Supplement Table S1**: Transcripts induced in human monocytes by B. malayi crude extract stimulation in comparison to unstimulated controls.

| *ID/Transcript* | *Gene* | *Definition* | *Nom. P.Value* | *FC* |
| --- | --- | --- | --- | --- |
| ILMN_1715401 | **MT1G** | metallothionein 1G | 2,03E-05 | 3,04 |
| ILMN_2124802 | **MT1H** | metallothionein 1H | 3,10E-05 | 2,99 |
| ILMN_1718766 | **MT1F** | metallothionein 1F | 3,16E-05 | 2,43 |
| ILMN_2173611 | **MT1E** | metallothionein 1E | 0,0004463 | 2,32 |
| ILMN_1686664 | **MT2A** | metallothionein 2A | 0,00071025 | 2,14 |
| ILMN_1691156 | **MT1A** | metallothionein 1A | 0,00068149 | 2,09 |
| ILMN_1775170 | **MT1X** | metallothionein 1X | 0,00060765 | 2,05 |
| ILMN_1656310 | **INDO** | indoleamine 2,3-dioxygenase 1 | 0,01480863 | 1,96 |
| ILMN_1657435 | **MT1M** | metallothionein 1M | 0,00474874 | 1,94 |
| ILMN_3239965 | **IDO1** | indoleamine 2,3-dioxygenase 1 | 0,02005119 | 1,91 |
| ILMN_1686109 | **CCL23** | C-C motif chemokine ligand 23 | 0,00030124 | 1,77 |
| ILMN_1769388 | **GJB2** | gap junction protein beta 2 | 6,09E-05 | 1,75 |
| ILMN_3237878 | **LOC100133875** | similar to Leukocyte immunoglobulin-like receptor, subfamily A (without TM domain), member 3 | 0,01584309 | 1,68 |
| ILMN_1785732 | **TNFAIP6** | TNF alpha induced protein 6 | 0,00256657 | 1,68 |
| ILMN_1786303 | **LILRA3** | leukocyte immunoglobulin like receptor A3 | 0,01752226 | 1,66 |
| ILMN_1776188 | **MAP1LC3A** | microtubule associated protein 1 light chain 3 alpha | 0,00252913 | 1,61 |
| ILMN_1662640 | **C20orf127/MT1P3** | metallothionein 1 pseudogene 3 | 0,00360858 | 1,61 |
| ILMN_3307868 | **CHI3L1** | chitinase 3 like 1 | 0,00016417 | 1,61 |
| ILMN_1764030 | **CCL23** | C-C motif chemokine ligand 23 | 0,00169947 | 1,56 |
| ILMN_1691364 | **STAT1** | signal transducer and activator of transcription 1 | 0,02646289 | 1,56 |
| ILMN_1690105 | **STAT1** | signal transducer and activator of transcription | 0,03108525 | 1,54 |
| ILMN_1661631 | **LILRA3** | leukocyte immunoglobulin like receptor A3 | 0,04043949 | 1,54 |
| ILMN_1695316 | **SLC39A8** | solute carrier family 39 member 8 | 0,00504967 | 1,51 |
| ILMN_2184373 | **IL8 / CXCL8** | interleukin 8, C-X-C motif chemokine ligand 8 | 0,00468001 | 1,50 |
| ILMN_1713832 | **SNORD15B** | small nucleolar RNA, C/D box 15B | 0,0015155 | -1,51 |
| ILMN_2082762 | **SNORD68** | small nucleolar RNA, C/D box 68 | 0,00135061 | -1,52 |
| ILMN_3309759 | **VTRNA1-1** | vault RNA 1-1 | 0,00228141 | -1,52 |
| ILMN_1684982 | **PDK4** | pyruvate dehydrogenase kinase 4 | 7,96E-06 | -1,53 |
| ILMN_3244640 | **SNORD96A** | small nucleolar RNA, C/D box 96A | 0,00084078 | -1,57 |
| ILMN_3249167 | **SNORA63** | small nucleolar RNA, H/ACA box 63 | 0,00244951 | -1,59 |
| ILMN_3240594 | **RNU4ATAC** | RNA, U4atac small nuclear (U12-dependent splicing) | 0,00561025 | -1,59 |
| ILMN_2208903 | **CD52** | CD52 molecule | 0,00280334 | -1,62 |
| ILMN_1782939 | **ALB** | albumin | 0,00672508 | -1,62 |
| ILMN_3238435 | **SNORA12** | small nucleolar RNA, H/ACA box 12 | 0,02534893 | -1,63 |
| ILMN_1901419 | **RNU11** | RNU11, RNA, U11 small nuclear | 0,00292359 | -1,64 |
| ILMN_3246209 | **SCARNA10** | small Cajal body-specific RNA 10 | 0,00383962 | -1,66 |
| ILMN_1908824 | **RNU6ATAC** | RNA, U6atac small nuclear (U12-dependent splicing) | 0,01291778 | -1,66 |
| ILMN_3241878 | **RNU6ATAC** | RNA, U6atac small nuclear (U12-dependent splicing) | 0,00870081 | -1,68 |
| ILMN_1704056 | **RPPH1** | ribonuclease P RNA component H1 | 0,00643212 | -1,75 |
| ILMN_3245103 | **RNU11** | RNA, U11 small nuclear | 0,00188116 | -1,78 |
| ILMN_1768139 | **RNU12** | RNA, U12 small nuclear | 0,00262698 | -1,78 |
| ILMN_3242405 | **RMRP** | RNA component of mitochondrial RNA processing endoribonuclease | 0,00048553 | -1,81 |
| ILMN_1688423 | **FCER1A** | Fc fragment of IgE receptor Ia | 8,21E-05 | -1,82 |
| ILMN_1682402 | **SNORD46** | small nucleolar RNA, C/D box 46 | 0,00268996 | -1,89 |

In total 42 transcripts were significantly (P value < 0.05; FC ≥ 1.5) induced in human monocytes (n=20 donors) after 24 h of *B. malayi* crude extract (BmA) stimulation compared to unstimulated controls.

**Suppl. Table S3**: BmA followed by LPS re-stimulation-induced transcripts in human monocytes in comparison to LPS-only stimulated controls.

| *ID/Transcript* | *Symbol* | *Definition* | *adj.P.Val* | *FC* |
| --- | --- | --- | --- | --- |
| ILMN_2171384 | **CXCL5** | C-X-C motif chemokine ligand 5, ENA-78, SCYB5 | 0,16580403 | 2,56 |
| ILMN_2124802 | **MT1H** | metallothionein 1H | 0,21755233 | 2,10 |
| ILMN_1752562 | **CXCL5** | C-X-C motif chemokine ligand 5, ENA-78, SCYB5 | 0,19664078 | 2,08 |
| ILMN_1767281 | **PPBP** | pro-platelet basic protein, CXCL7; NAP-2 | 0,24213693 | 1,90 |
| ILMN_1657435 | **MT1M** | metallothionein 1M | 0,36481335 | 1,80 |
| ILMN_1718766 | **MT1F** | metallothionein 1F | 0,31856643 | 1,74 |
| ILMN_1796316 | **MMP9** | matrix metallopeptidase 9, | 0,07680070 | 1,70 |
| ILMN_1715401 | **MT1G** | metallothionein 1G | 0,51871992 | 1,70 |
| ILMN_1779234 | **CXCL6** | C-X-C motif chemokine ligand 6, GCP2 | 0,10022298 | 1,62 |
| ILMN_1709204 | **CLEC4A** | C-type lectin domain family 4 member A, CD367 | 0,14503500 | 1,58 |
| ILMN_1733579 | **EVI2A** | ecotropic viral integration site 2A | 0,18209529 | 1,57 |
| ILMN_1780671 | **PLEKHG3** | pleckstrin homology and RhoGEF domain containing G3 | 0,13891895 | 1,56 |
| ILMN_1690342 | **LTA4H** | leukotriene A4 hydrolase | 0,37794065 | 1,55 |
| ILMN_2161577 | **CXCL6** | C-X-C motif chemokine ligand 6, GCP2 | 0,07680070 | 1,55 |
| ILMN_1740015 | **CD14** | CD14 molecule | 0,45457167 | 1,55 |
| ILMN_1689518 | **PECAM1** | platelet and endothelial cell adhesion molecule 1 | 0,12248916 | 1,54 |
| ILMN_1735910 | **VMO1** | vitelline membrane outer layer 1 homolog | 0,33937674 | 1,53 |
| ILMN_1763452 | **EVI2B** | ecotropic viral integration site 2B | 0,13891895 | 1,53 |
| ILMN_1690125 | **PDLIM7** | PDZ and LIM domain 7 | 0,06134147 | 1,52 |
| ILMN_3246869 | **SCARNA21** | small Cajal body-specific RNA 21 | 0,12483774 | -1,51 |
| ILMN_2208903 | **CD52** | CD52 molecule | 0,41519934 | -1,51 |
| ILMN_1801842 | **PTX3** | pentraxin 3, TSG-14; TNFAIP5 | 0,03244278 | -1,53 |
| ILMN_1691846 | **G0S2** | G0/G1 switch 2 | 0,12483774 | -1,53 |
| ILMN_3241021 | **RNY4** | RNA, Ro-associated Y4 | 0,27347828 | -1,54 |
| ILMN_3236049 | **SNORD12** | small nucleolar RNA, C/D box 12 | 0,14863846 | -1,54 |
| ILMN_1704730 | **CD93** | CD93 molecule | 0,45819779 | -1,57 |
| ILMN_1689378 | **CCRN4L** | CCR4 carbon catabolite repression 4-like | 0,07680070 | -1,57 |
| ILMN_1699695 | **TNFRSF21** | TNF receptor superfamily member 21 | 0,19664078 | -1,58 |
| ILMN_2082762 | **SNORD68** | small nucleolar RNA, C/D box 68 | 0,13891895 | -1,60 |
| ILMN_1810462 | **C10orf115** | chromosome 10 open reading frame 115 / Ba215c7.4 | 0,14863846 | -1,60 |
| ILMN_3244640 | **SNORD96A** | small nucleolar RNA, C/D box 96A | 0,14503500 | -1,62 |
| ILMN_3309759 | **VTRNA1-1** | vault RNA 1-1 | 0,14863846 | -1,62 |
| ILMN_1713832 | **SNORD15B** | small nucleolar RNA, C/D box 15B | 0,12972666 | -1,63 |
| ILMN_3240594 | **RNU4ATAC** | RNA, U4atac small nuclear (U12-dependent splicing) | 0,24872237 | -1,65 |
| ILMN_1713751 | **ADAM19** | ADAM metallopeptidase domain 19 | 0,30719616 | -1,69 |
| ILMN_3249167 | **SNORA63** | small nucleolar RNA, H/ACA box 63 | 0,13849379 | -1,73 |
| ILMN_1657234 | **CCL20** | C-C motif chemokine ligand 20 | 0,31270144 | -1,74 |
| ILMN_1737709 | **RPL10L** | ribosomal protein L10 like | 0,07680070 | -1,76 |
| ILMN_3246209 | **SCARNA10** | small Cajal body-specific RNA 10 | 0,17434420 | -1,78 |
| ILMN_1901419 | **RNU11** | RNA, U11 small nuclear | 0,13891895 | -1,79 |
| ILMN_3242405 | **RMRP** | RNA component of mitochondrial RNA processing endoribonuclease | 0,10022298 | -1,90 |
| ILMN_3241878 | **RNU6ATAC** | RNA, U6atac small nuclear (U12-dependent splicing) | 0,17434420 | -1,91 |
| ILMN_3245103 | **RNU11** | RNA, U11 small nuclear | 0,13849379 | -1,91 |
| ILMN_3238435 | **SNORA12** | small nucleolar RNA, H/ACA box 12 | 0,22461734 | -1,91 |
| ILMN_1704056 | **RPPH1** | ribonuclease P RNA component H1 | 0,18209529 | -1,92 |
| ILMN_1908824 | **RNU6ATAC** | RNA, U6atac small nuclear (U12-dependent splicing) | 0,18003597 | -1,94 |
| ILMN_1782939 | **ALB** | albumin | 0,10022298 | -1,96 |
| ILMN_1768139 | **RNU12** | RNA, U12 small nuclear | 0,12248916 | -2,00 |
| ILMN_1682402 | **SNORD46** | small nucleolar RNA, C/D box 46 | 0,12248916 | -2,11 |

In total 49 transcripts were significantly (P value < 0.05; FC ≥ 1.5) induced in human monocytes (n=20 donors) after 18 h of *B. malayi* crude extract (BmA) priming followed by 6h of LPS re-stimulation in comparison to LPS-only stimulated controls (BmA+LPS vs. LPS).

**Suppl. Table S4:** Canonical Pathways analysis of BmA primed and LPS re-stimulated human monocytes.

| Ingenuity Canonical Pathways | -log(p-value) | Molecules |
| --- | --- | --- |
| Granulocyte Adhesion and Diapedesis | 6,74E00 | PPBP, PECAM1, *CCL20*, **CXCL5**, *CXCL6*, *MMP9* |
| Agranulocyte Adhesion and Diapedesis | 6,57E00 | PPBP, PECAM1, *CCL20*, **CXCL5**, *CXCL6*, *MMP9* |
| Role of IL-17A in Psoriasis | 6,1E00 | *CCL20*, **CXCL5**, *CXCL6* |
| Role of IL-17A in Arthritis | 4,18E00 | *CCL20*, **CXCL5**, *CXCL6* |
| IL-17A Signaling in Airway Cells | 3,96E00 | *CCL20*, **CXCL5**, *CXCL6* |
| LXR/RXR Activation | 3,15E00 | ALB, CD14, *MMP9* |
| Role of IL-17F in Allergic Inflammatory Airway Diseases | 2,73E00 | **CXCL5**, *CXCL6* |
| Atherosclerosis Signaling | 1,85E00 | ALB, *MMP9* |
| Hepatic Fibrosis / Hepatic Stellate Cell Activation | 1,54E00 | CD14, *MMP9* |
| Leukocyte Extravasation Signaling | 1,48E00 | PECAM1, *MMP9* |
| Axonal Guidance Signaling | 8,83E-01 | ADAM19, *MMP9* |

Canonical Pathways identified by Ingenuity Pathways Knowledge Base analyzing human monocytes after 18h treatment with BmA followed by 6h LPS stimulation compared to LPS alone (BmA+LPS vs. LPS). Transcripts that were confirmed by qPCR or ELISA with statistical significance are marked as bold and by trend as italics. P-value and affected molecules (n≥2) are presented.
